# A rare variant in the FHL1 gene associated with X-linked recessive hypoparathyroidism

**DOI:** 10.1007/s00439-017-1804-9

**Published:** 2017-04-25

**Authors:** Nir Pillar, Oren Pleniceanu, Mingyan Fang, Limor Ziv, Einat Lahav, Shay Botchan, Le Cheng, Benjamin Dekel, Noam Shomron

**Affiliations:** 1 Sackler Faculty of Medicine, Tel Aviv University, Tel Aviv, Israel; 2 Pediatric Stem Cell Research Institute & Division of Pediatric Nephrology, Edmond & Lily Safra Children’s Hospital, Sheba Medical Center, Tel Hashomer, Israel; 3 BGI-Yunnan, Kunming, China; 4 Sheba Cancer Research Center, Sheba Medical Center, Tel Hashomer, Israel

## Abstract

Isolated familial hypoparathyroidism is an extremely rare disorder, which to date has been linked to several loci including mutations in *CASR*, *GCM2,* and *PTH*, as well as a rare condition defined as X-linked recessive hypoparathyroidism, previously associated with a 1.5 Mb region on Xq26-q27. Here, we report a patient with hypocalcemia-induced seizures leading to the diagnosis of primary hypoparathyroidism. Mutations in *CASR*, *GCM2,* and *PTH* were ruled out, while whole exome sequencing of the family suggested *FHL1*, located on chromosome Xq26, as the most likely causative gene variant (*FHL1*, exon 4, c.C283T, p.R95W). Since FHL1 has not been linked to calcium regulation before, we provide evidence for its functional role in hypoparathyroidism by: (i) bioinformatics analysis coupling its action to known modulators of PTH function; (ii) observing strong expression of *fhl1b* in Corpuscles of Stannius, gland-like aggregates in zebrafish that function in calcium regulation similar to mammalian PTH; and (iii) implicating fhl1b and FHL1 as regulators of calcium homeostasis in zebrafish and human cells, respectively. Altogether, our data suggest that FHL1 is a novel regulator of calcium homeostasis and implicate it as the causative gene for X-linked recessive hypoparathyroidism.

**Electronic supplementary material:**

The online version of this article (doi:10.1007/s00439-017-1804-9) contains supplementary material, which is available to authorized users.

## Introduction

**Fig. 1:**
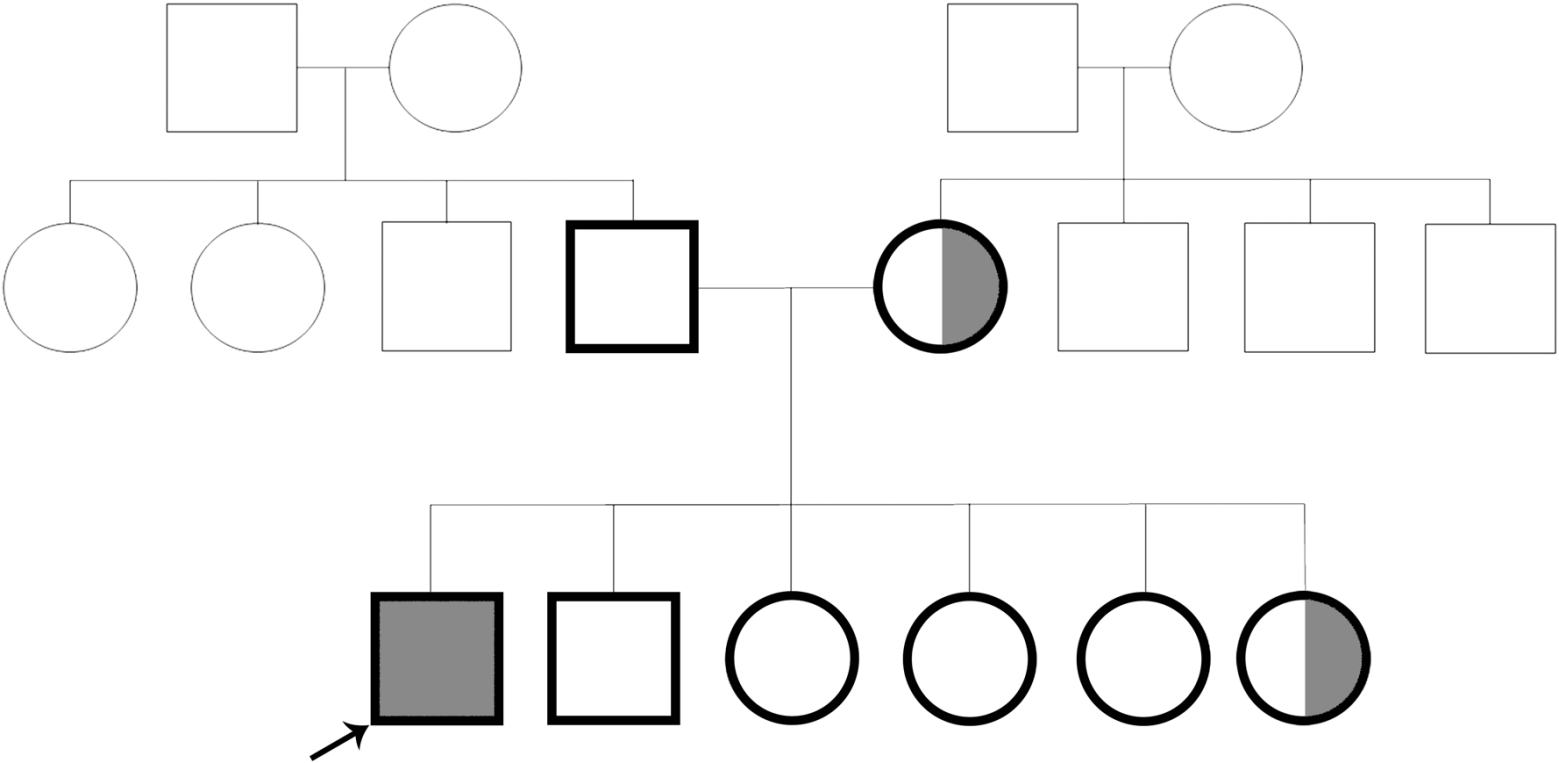
Family pedigree. *Squares* denote male family members, *circles* female members, and *shaded symbols* affected members; the *arrow* points to the proband

Deep Sequencing, or Next Generation Sequencing (NGS), is a powerful tool to identify causative variants in clinical cases, where other methods have been exhausted. Here, we describe a 4-year-old male, born at full term following an uneventful pregnancy and delivery to non-consanguineous parents, and four siblings (Fig. 1) presented with generalized seizure at the age of 9 days. Upon ruling out all other major etiologies for seizures, initial evaluation identified severe hypocalcemia of 5.7 mg/dl [9–11], hyperphosphatemia of 11.5 mg/dl [5–9], and inappropriately low PTH of 20.7, alongside normal albumin, magnesium, potassium, chloride, blood gases, and vitamin D levels. Urinary calcium levels were low at 0.18 mg/dl [5.2–35.7]. Physical examination revealed no dysmorphic features, short stature, malformations, or developmental delay. Additional studies included chest X-ray, brain US, echocardiogram, renal ultrasound, electroencephalogram, bone age study, and audiogram were all normal. Following a clinical geneticist consultation, 22q11.2 deletion was ruled out via Fluorescent In-Situ Hybridization (FISH). Sanger Sequencing detected no genomic variants in *PTH* and *GCM2* genes. Since no additional loci were related to the phenotype, array-CGH was not performed.

At the age of 4 years, the patient continued to exhibit persistent hypocalcemia alongside inappropriately low PTH levels. In addition, mild orolingual muscle weakness was detected in the patient. Notably, a younger, sixth female sibling was also found to have mild hypoparathyroidism albeit without any electrolyte abnormality, while all other family members exhibit normal PTH and calcium levels. The patient’s father, as well as all other male siblings, demonstrates slight hypotonicity, requiring occupational therapy.

Taken together, these findings indicate that the patient suffers from isolated familial hypoparathyroidism of unknown origin. Exome sequencing of the affected male, his parents, and siblings was performed to detect a causal gene.

## Results

### Genomic analysis uncovers *FHL1* as the causative gene

Whole exome sequencing (WES) of the patient generated 1,065,106 variants passing initial filters, 58,977 of which were rare variants (allele frequency <1% in all databases), and 1031 were found inside exons and resulted in an amino acid change. When searching for de novo mutations found in the patient or in his parents/siblings, only one variant with low allele coverage (<5×) was detected. When focusing on homozygous recessive/X-linked variants, four variants remained. Prioritization of these variants by combining variant severity and gene information revealed an X-linked nonsynonymous mutation in *FHL1*, ‘Four and a Half LIM domains 1’ gene (*FHL1*, exon 4, c.C283T, p.R95W) to be the top candidate variant. The variant was predicted to be deleterious by the highest number of prediction tools (8.5/10; see “Materials and methods”). It is a very rare variant with prevalence of 0.0005/45 in the Exome Aggregation Consortium, 0.0006/6 in the NHLBI exome sequencing project and is not present in the 1000 genomes project, or is it found in our personal database of over 900 Israeli exomes. Sanger sequencing confirmed the variant is present in the patient, heterozygous in his mother, and not detected in other family members.

The *FHL1* c.C283T variant was predicted by the majority of our employed tools to be deleterious, it is found in a conserved region of the gene, and alternate allele coverage was >30×. Manually reviewing the three other homozygous recessive/X-linked variants (*ZNF366* c.A58G p.K20E, *CORO1B* c.G1252A p.A418T, and *ZNF208* c.T986C p.I329T), all three variants were not predicted to be deleterious by any of the tools we employed.

**Fig. 2:**
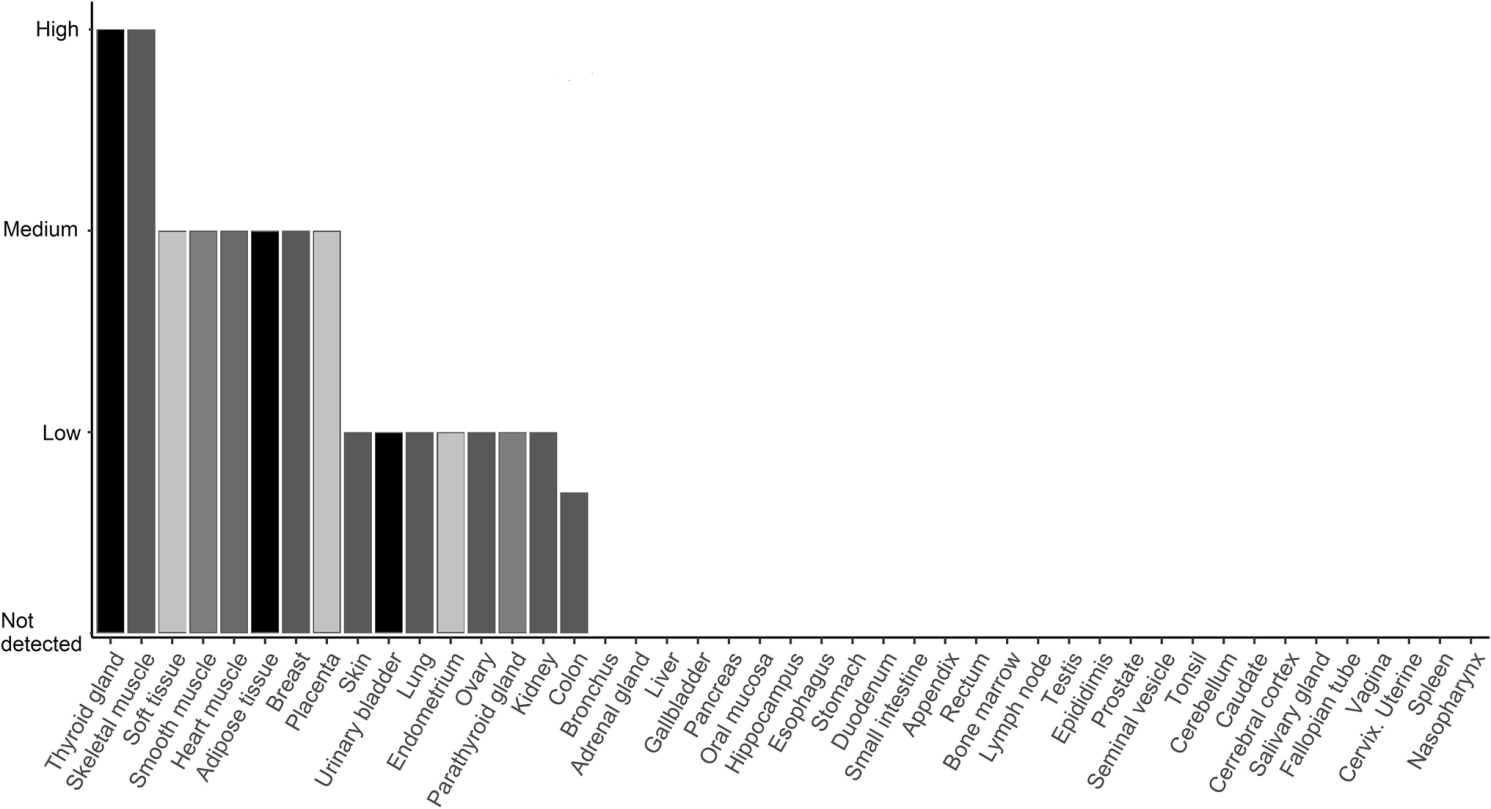
Expression of FHL1 protein in human tissues, estimated by immunohistochemistry (IHC) using FHL1 antibody. Height of the bars represents IHC staining strength. Taken from the Human Protein Atlas (Uhlén et al. 2015)

*FHL1* (also known as *SLIM*, *SLIM1,* or *SLIMMER*) is a member of the gene family encoding LIM domain containing proteins. An LIM domain is mainly constituted of two cysteine-rich zinc-finger motifs, which coordinately bind zinc atoms to mediate protein–protein interactions (Kadrmas and Beckerle 2004). FHL1 expression is highly enriched in striated muscles (Fig. 2), and has, therefore, been suggested to play an important role in skeletal muscle growth and remodeling (Cowling et al. 2008). FHL1 was demonstrated to have an important role in muscle development and disease. A number of genetic studies linked *FHL1* missense mutations to congenital myopathies previously recognized by particular structural features, including Reducing body myopathy (RBM) and Emery-Dreifuss Muscular Dystrophy (EDMD) (Cowling et al. 2011). Mutations in the *FHL1* gene have also been associated with arrhythmias (San Román et al. 2016), HCM (Xu et al. 2015), and dilated cardiomyopathy (Christodoulou et al. 2014) in several patients affected by skeletal muscle disorders as well.

Although the patient exhibits minimal muscular symptoms (see above), the phenotype differs significantly than previously ascribed to *FHL1* mutations. Nonetheless, due to the clinical assessment that the patient’s severe phenotype and early onset strongly suggest a genetic cause, we have decided to further pursue the connection between *FHL1* and hypocalcemia.

### FHL1 interacts with calcium-regulatory proteins

Using gene ontology, we tested the biological pathways that were enriched by some of the 11 core genes plus *FHL1*. Several pathways associated with molecules transport, including ion transport, transmembrane transport, and regulation of transport, were enriched with at least four of the manually curated targets plus FHL1 (Supplementary Table 1). Next, to test the interactions between FHL1 and hypocalcemia-related proteins, we have manually assembled a list of 11 genes *(PTH, CALC1, PTHRP, TBX1, GCM2, CASR, AIRE, GNA11, GATA3, GNAS,* and *TRPM6*), which code for proteins known to be involved in calcium sensing/metabolism with direct connection to primary hypoparathyroidism.

**Fig. 3:**
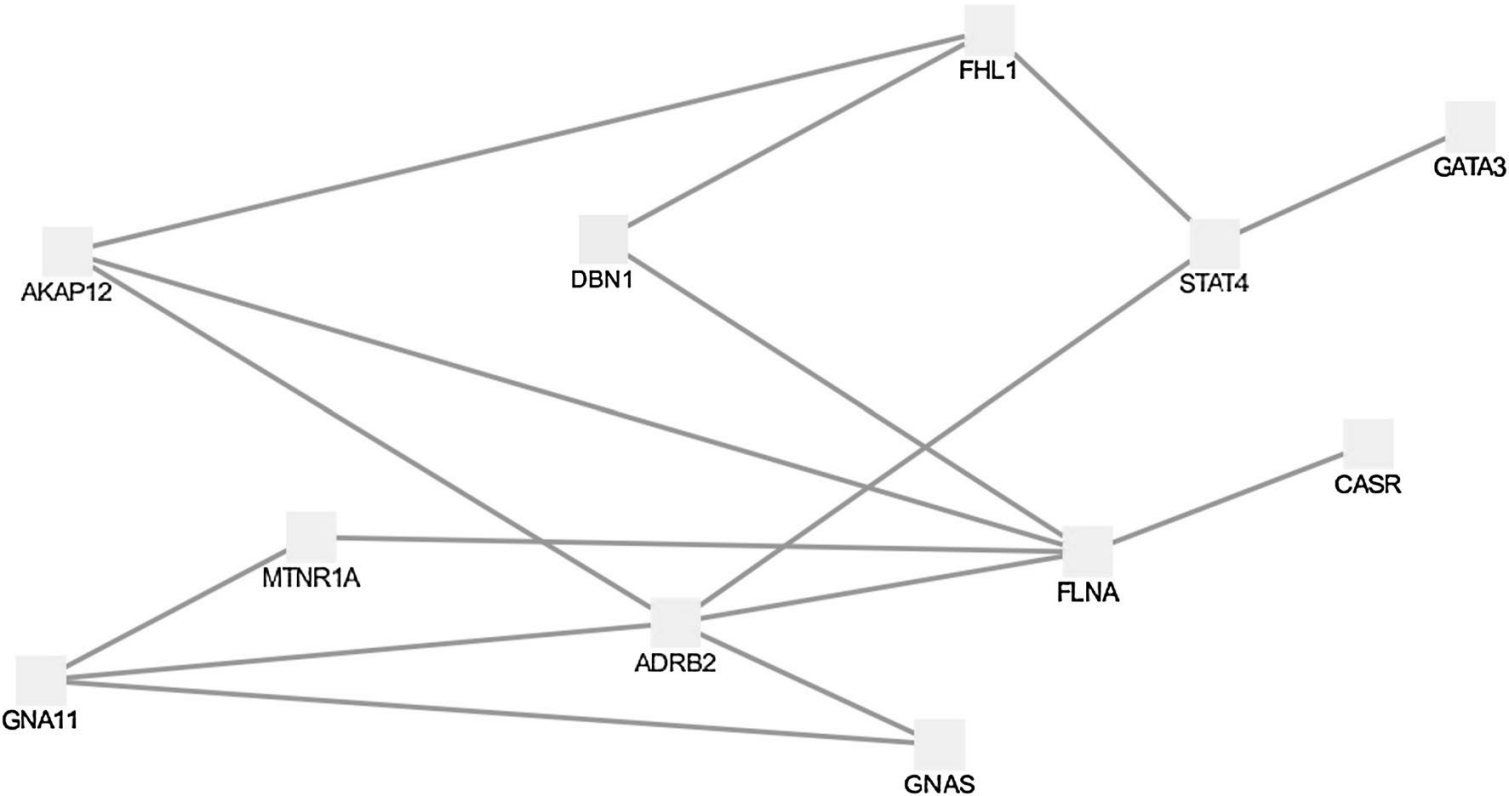
Induced module of FHL1 and connected proteins of interest. Nodes with *black labels* are seed proteins, and nodes with *gray labels *are intermediate nodes. Taken from ConsensusPathDB (Kamburov et al. 2011)

We have assembled an induced network module, consisting of the 11 proteins coded by hypocalcemia-related genes and FHL1 (Fig. 3). The induced network demonstrated an indirect connection between FHL1 and GATA3 (via STAT4) and CASR (via AKAP12 and FLNA).

STAT4 is a transcription factor belonging to the STAT protein family that is expressed mostly in immune cells and binds to hundreds of sites in the genome. It was shown that FHl1 promotes both the degradation and the dephosphorylation of STAT4 (Tanaka et al. 2005), presumably affecting GATA3 expression via this mechanism.

AKAP12 (A-kinase anchor proteins) is a member of a structurally diverse protein group, which has the common function of binding to the regulatory subunit of protein kinase A (PKA) and confining the holoenzyme to discrete locations within the cell. A connection with FHL1 was noted in direct protein interaction (Vinayagam et al. 2011) and FLNA (Malovannaya et al. 2011). FLNA, coded for Filamin A protein, is known to interact with CASR (calcium-sensing receptor) and likely act as part of a scaffold that binds signaling components and other key cellular elements (e.g., the cytoskeleton) to facilitate the interaction of the receptor with its signaling pathways (Ray 2015).

### *fhl1b* regulates calcium levels in zebrafish

The zebrafish (Danio rerio) is a powerful model organism for studying vertebrate biology, being well suited to both developmental and genetic analysis (Dooley and Zon 2000). Regulation of Ca^2+^ levels in vertebrates requires the ability to sense extracellular Ca2^+^ concentrations, a process carried out by the transmembrane calcium-sensing receptor (CASR) (Loretz 2008). Low Ca^2+^ levels sensed by CASR in the parathyroid gland, the main organ responsible for calcium homeostasis, lead to expression and secretion of PTH, the key hypercalcemic hormone, which acts on the kidneys, bones, and intestine to increase calcium levels. In contrast, lower vertebrates, like fish rely mostly on gills (or skin, during embryonic stages) as the primary organs for Ca^2+^ uptake (Hwang et al. 2011; Liao et al. 2007). Nonetheless, although fish do not harbor parathyroid glands, similar calcium-regulating pathways have been uncovered, involving mainly the Corpuscles of Stannius (CS), gland-like aggregates adjacent to the fish kidneys. CS strongly express CASR, which mediates the expression of PTH1 and STC1 (Stanniocalcin), the main hypercalcemic, and hypocalcemic hormones in fish, respectively (Hwang and Chou 2013). Thus, zebrafish represent an important model for studying calcium homeostasis in both physiological and disease states.

Similar to many other genes, due to teleost genome duplication, *FHL1* is represented in fish by two paralogs: *fhl1a* and *fhl1b* (Glasauer and Neuhauss 2014). In light of its human phenotype, most studies to date have focused on the role of *fhl1a* and *fhl1b* on muscular and cardiac function (Bührdel et al. 2015; Chauvigné et al. 2005; Li et al. 2013) and more recently (Xu et al. 2016) on its role in pancreas-liver fate decision during development. Accordingly, in-situ hybridization experiments have demonstrated (Thisse et al. 2015) expression of *fhl1a* and *fhl1b* in the zebrafish muscular system, heart and pancreas, among others. Interestingly, *fhl1b* has been shown to be expressed strongly and specifically in the CS. Hence, we hypothesized that *fhl1b* might be involved in calcium regulation in fish.

To test this hypothesis, we injected fish embryos with morpholinos to inhibit the translation of zebrafish *fhl1b* and compared free plasma calcium levels between morpholinos-injected (*fhl1b* ATG MO) and uninjected (UI) fish. *fhl1b* expression was decreased by 45% in ATG MO group (SEM—3.2%, *P* < 0.01). The analysis was carried out in fish grown in media containing both low and high physiologic (25 μg/ml) calcium levels, so as to evaluate the potential relevance of *fhl1b* to calcium homeostasis across a wide range of calcium levels.

**Fig. 4:**
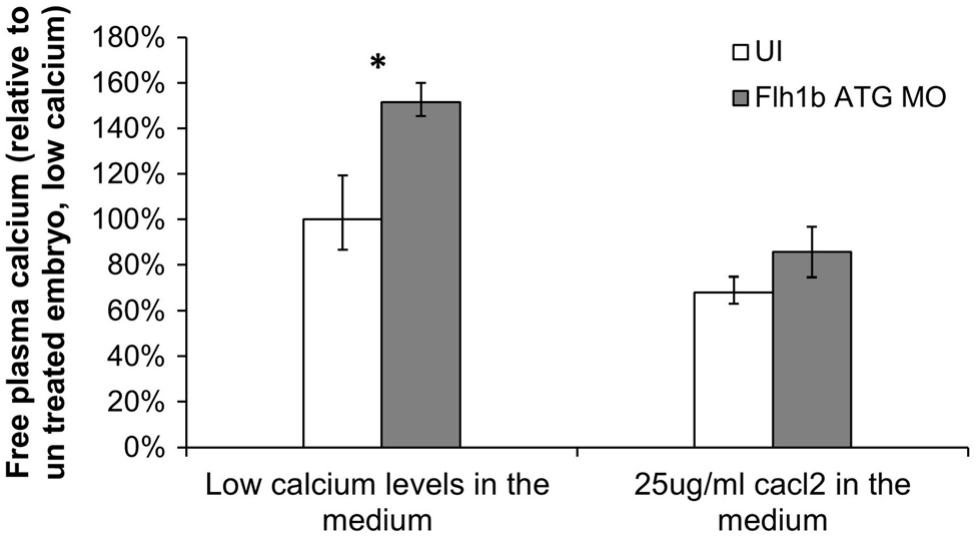
Dysregulation of *Fhl1b* KD on calcium levels. *Fhl1b* ATG MO was used for Fhl1b KD. Calcium levels were measured in larvae with *fhl1b* KD vs. UI and in low calcium medium levels vs. 25 μg/ml CaCl_2_ medium levels (high physiologic calcium level). Data show the mean ± SEM (*n*  =  3). *P < 0.05

We detected an increase in plasma calcium levels in *fhl1b* ATG MO fish compared UI fish in both low and high calcium media (*P* < 0.05 for fish grown in low calcium media and *P* = 0.12 for 25 μg/ml Calcium media) (Fig. 4). Taken together, these results indicate a role for *fhl1b* in regulating fish plasma calcium levels.

### *fhl1b* expression affects other calcium-regulatory genes

**Fig. 5:**
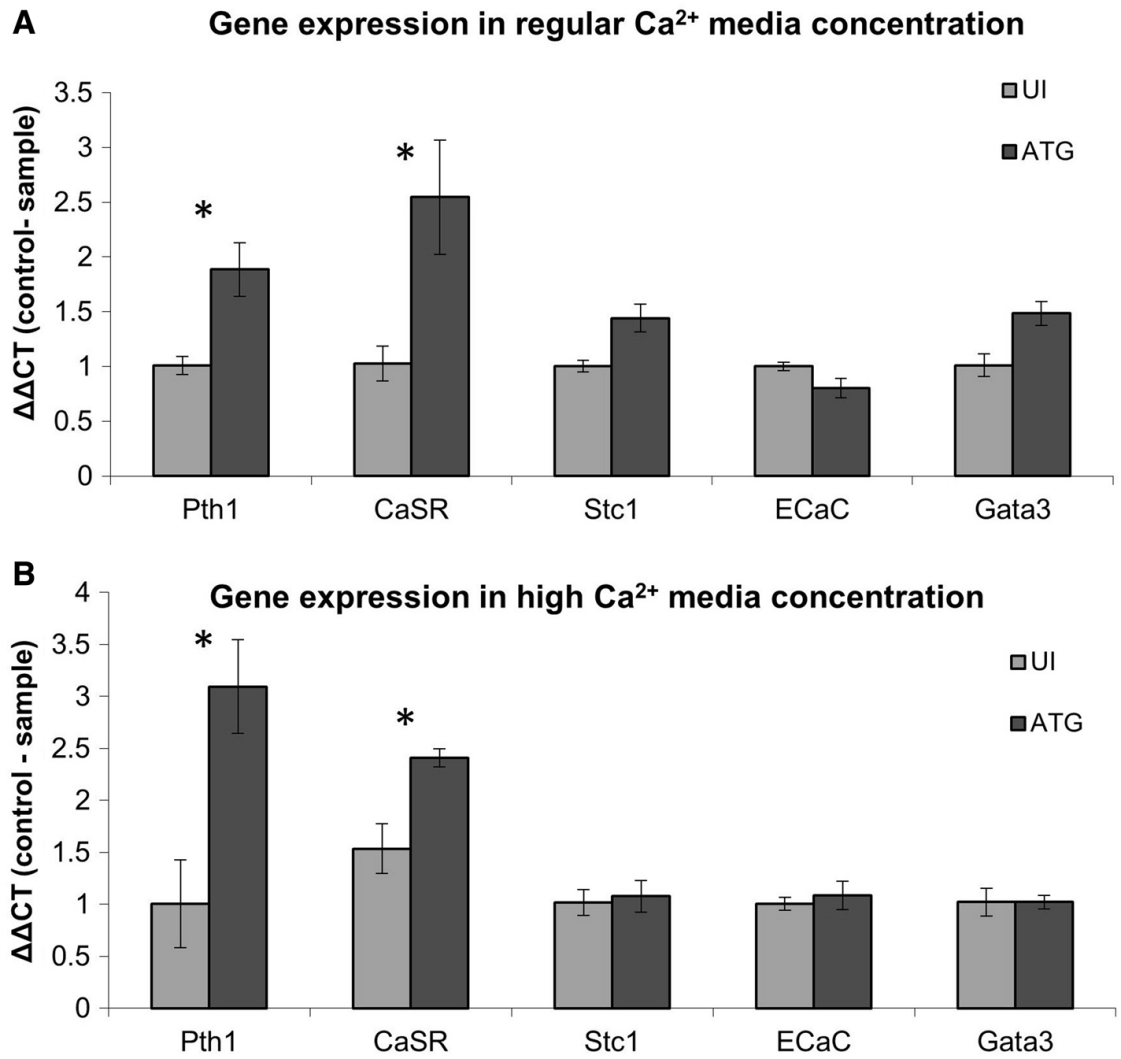
Regulation of several calcium homeostasis-related genes by *fhl1b* KD. Fish were injected with *fhl1b* MO and were exposed to regular water (**a**)/high Ca^2+^ water levels (**b**). RT-PCR was used for expression level calculation of parathyroid hormone paralog 1 (PTH1), calcium-sensing receptor (CASR), Stanniocalcin (Stc1), epithelial calcium channel (ECaC), and GATA Binding Protein 3 (Gata3). Beta-actin expression was used as control gene. Data show the mean ± SEM (*n*  =  3). *P < 0.05

Having demonstrated the significant effect of *fhl1b* down-regulation on zebrafish plasma calcium levels, we were next interested in determining the mediators of this effect. Towards this end, we compared the expression of key calcium-regulatory genes (parathyroid hormone paralog 1—PTH1, calcium-sensing receptor—CASR, Stanniocalcin—STC1, epithelial calcium channel—ECaC, and GATA Binding Protein 3-Gata3) in *fhl1b* ATG MO fish to UI fish via quantitative RT-PCR (qPCR) (Fig. 5). Consistent with the increased calcium levels observed in *fhl1b* ATG MO fish, we detected in the latter significant up-regulation of *PTH1*, the main hypercalcemic hormone in both fish and mammals, compared to UI fish. Interestingly, we also found induction of CASR transcript levels in *fhl1b* ATG MO fish. All other genes were not affected by *fhl1b* down-regulation (Fig. 5). Hence, increased calcium levels detected in fhl1b-deficient fish are associated with increased PTH1/CASR expression.

### *fhl1b* is a positive regulator of PTH expression in human cells

**Fig. 6:**
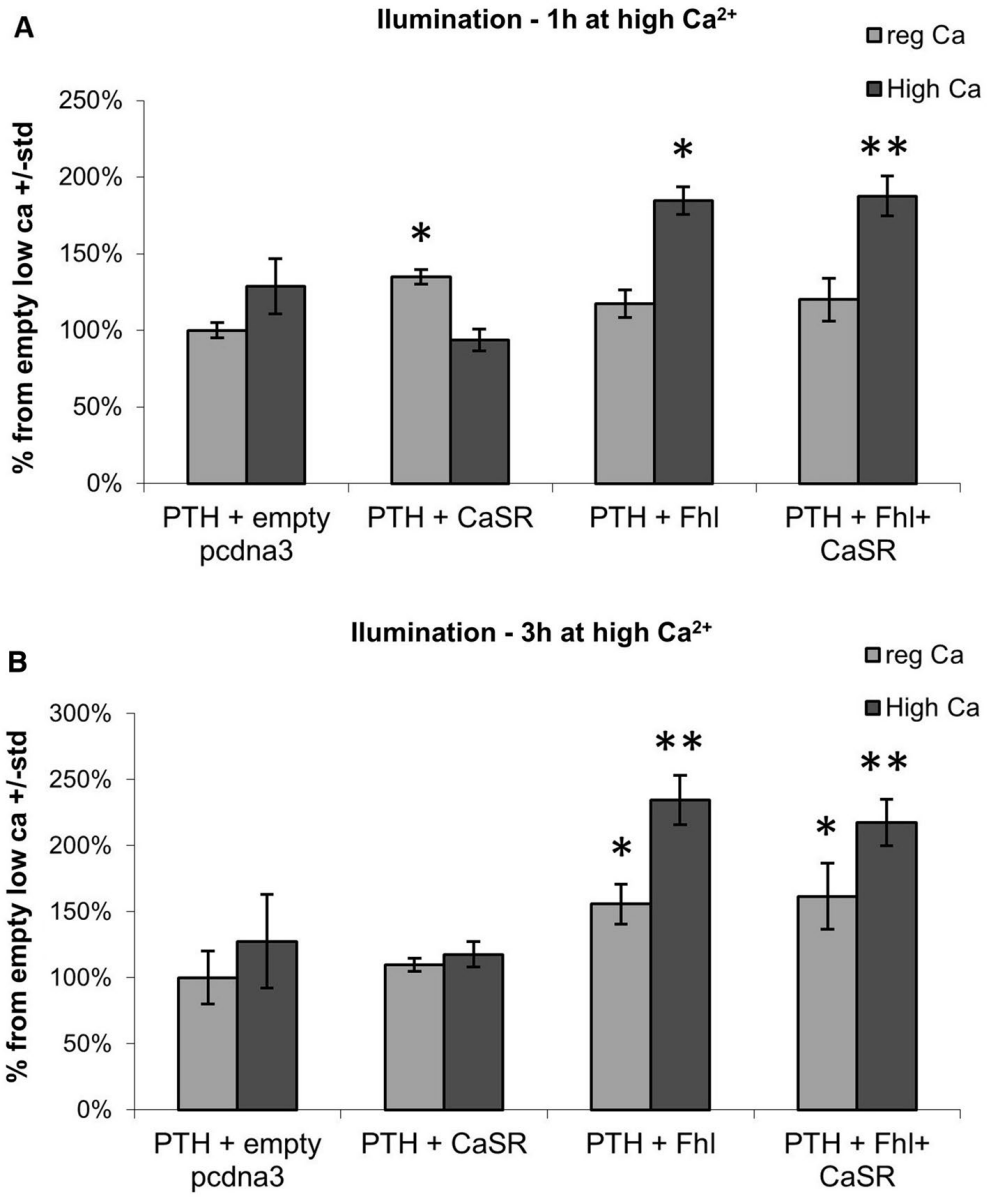
HEK293 cells were co-transfected with PTH under Luciferase promoter with co-expression of CASR and FHL1. Effect was measured as changes in luminescence. **a** Effect of CASR and FHL1 co-transfection on luminescence at 1 h of high Ca^2+^. **b** Effect of CASR and FHL co-transfection on luminescence at 3 h of high Ca^2+^. Data show the mean ± SEM (*n*  =  3). *P < 0.05, **P < 0.01

**Fig. 7:**
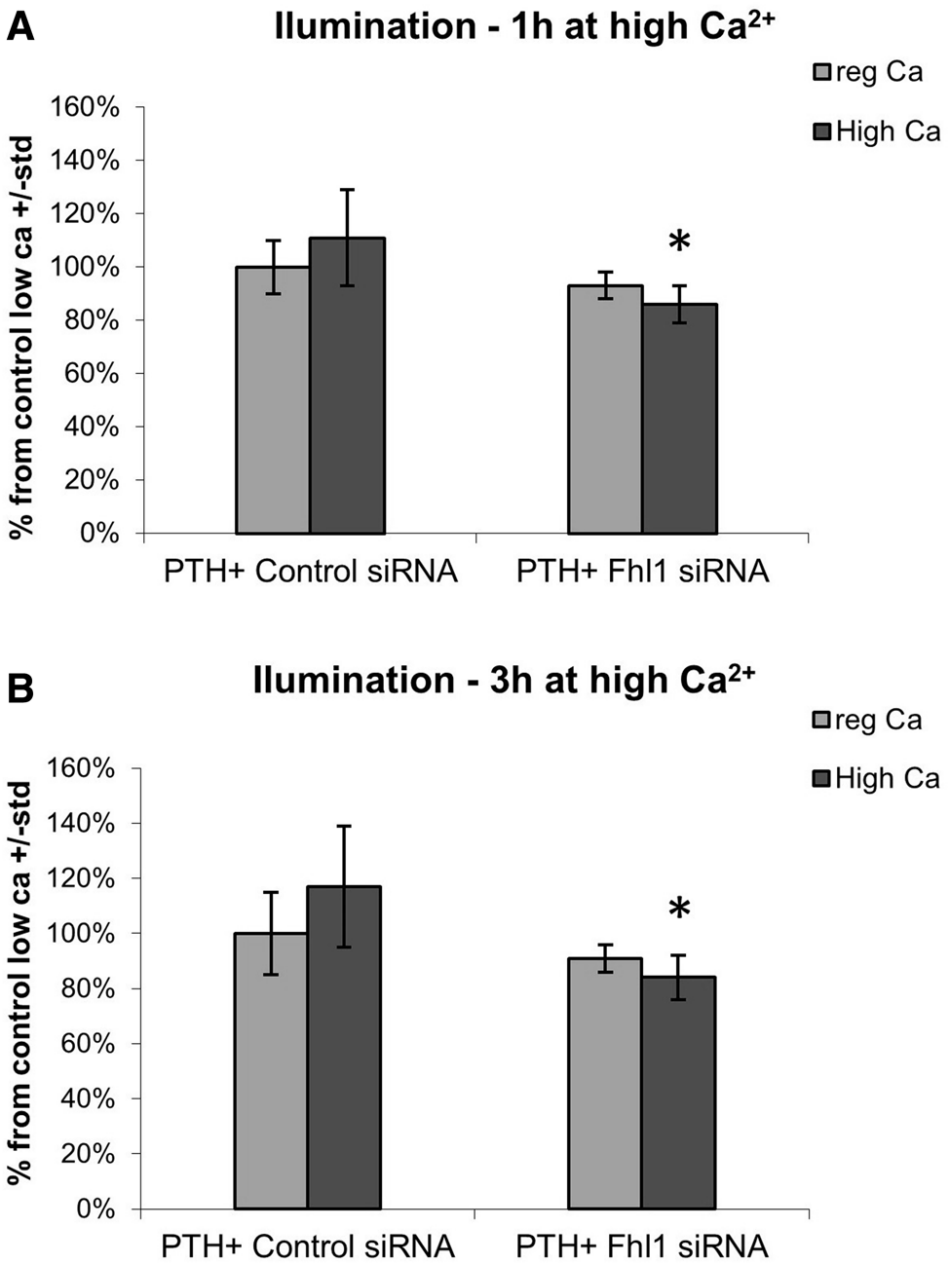
HEK293 cells were co-transfected with PTH under Luciferase promoter with siRNA against FHL1. Effect was measured as changes in luminescence. **a** Effect of FHL1 siRNA on luminescence at 1 h of high Ca^2+^. **b** Effect of FHL1 siRNA on luminescence at 3 h of high Ca^2+^. Data show the mean ± SEM (*n*  =  3). *P < 0.05

To assess a potential direct effect of FHL1 on calcium regulation via PTH expression in human cells, we used a luciferase reporter assay, whereby human embryonic kidney (HEK293) cells were transfected with a construct containing the luciferase reporter under the control of the human PTH promoter. Activation of PTH locus was measured by bioluminescence in the presence of empty vector or FHL1, with and without CASR presence, under both normal (1.8 mM calcium) and high calcium conditions (3.6 mM calcium). We detected a strong and significant increase in activation of the PTH promoter when FHL1 was introduced into the cells, in both normal and high calcium conditions (Fig. 6). Next, activation of PTH locus was measured by bioluminescence in the presence of siRNA against *FHL1* or control siRNA. A consistent decrease in PTH activation was noted in the *FHL1* siRNA group which was more significant in the high calcium conditions (Fig. 7). Taken together, these results indicate that FHL1 might function as a positive regulator of PTH expression in human cells.

## Conclusions

The human *FHL1* gene, located on chromosome Xq26, consists of eight exons, giving rise to three protein isoforms: FHL1A, FHL1B, and FHL1C. FHL1A is the predominant isoform in skeletal and cardiac muscle, and comprises an N-terminal half LIM domain followed by four complete LIM domains. FHL1B and FHL1C, the minor isoforms, share the same N-terminal two and a half LIM domains as FHL1A; however, alternative splicing results in unique C-termini: FHL1B contains nuclear import and export sequences and an RBP-J-binding domain, while the FHL1C C-terminus only consists of an RBP-J-binding domain (Cowling et al. 2011). The protein-binding LIM domains demonstrate a unique structure, consisting of highly conserved cysteine and histidine residues which bind Zn^2+^ and are required for fold and stability of each LIM domain structure. The FHL1 protein has an established role in mammalian skeletal muscle, where it localizes to the nucleus and focal adhesions and is able to shuttle between these compartments. FHL1A has been shown to play a role in promoting myoblast fusion and muscle growth via NFATc1 activation (Cowling et al. 2008).

Accordingly, a wide range of FHL1 mutations have been associated with various muscle- and cardiac-related disease phenotypes in humans. *FHL1* mutations were first reported in 2008 and linked to three phenotypes: X-linked myopathy with postural muscle atrophy and generalized hypertrophy (XMPMA),

X-linked dominant scapuloperoneal myopathy (X-SM), and classical reducing body myopathy (RBM) (Windpassinger et al. 2008; Schessl et al. 2008; Quinzii et al. 2008). Many reports later described additional mutations that broadened the phenotypic spectrum further, adding Emery–Dreifuss muscular dystrophy (EDMD) and hypertrophic cardiomyopathy (HCM) with no or minimal musculo-skeletal involvement to the clinical presentations of *FHL1* mutations (Gueneau et al. 2009; Friedrich et al. 2012).

Herein, we report a male patient, demonstrating primary hypoparathyroidism, manifesting in significant hypocalcemia from birth. Isolated familial hypoparathyroidism is an extremely rare disorder, which to date has been linked to four different loci. These include mutations in *CASR, GCM2,* and *PTH*, as well as a rare condition defined as “X-linked recessive hypoparathyroidism (XLHPT)” (Whyte and Weldon 1981; Winter et al. 1983). The latter condition was described in two multi-generation families, where affected males demonstrated neonatal idiopathic hypoparathyroidism with consequent hypocalcemia and were also sterile. Post-mortem analysis of one of the patients demonstrated aplasia of the parathyroid gland. Carrier females were normo-calcemic and asymptomatic. The XLHPT locus was mapped to a 906-kb region on Xq27 that contains three genes (*ATP11C, U7snRNA*, and *SOX3*) (Trump et al. 1998). STS and SNP-based analyses identified a 23–25-kb deletion, which did not contain genes. The deleted region was replaced by a 340-kb insertion that originated from 2p25 and contained a segment of the *SNTG2* gene that lacked an open reading frame. Hence, the authors speculated that this insertion could have a position effect on SOX3 expression, which was shown to be expressed in the developing mouse parathyroid between E10.5 and E15.5 (Bowl et al. 2005). Notably, however, *SOX3* mutations have never been reported to cause hypocalcemia nor have SOX3 been linked functionally to calcium homeostasis in any organism. Mutations in *CASR* were ruled out in our patient on a clinical basis (i.e., lack of calciuresis) and *GCM2* and *PTH* mutations were ruled out via Sanger sequencing. Therefore, we carried out WES of the family, which identified *FHL1* as the most likely causative gene. Although FHL1 has never been linked to calcium regulation, the strong bioinformatic evidence, the mild muscular phenotype that developed in the patient and the close proximity (~3 Mb) of the *FHL1* locus to the XLHPT locus, convinced us to pursuit a potential role for FHL1 in calcium homeostasis using the zebrafish model. While XLHPT prevalence is less than 1 in 1 000 000 live births, the *FHL1* c.C283T variant is reported in the Exome Aggregation Consortium with a prevalence of 0.0005. There are also 11 hemizygotes reported in the Exome Aggregation Consortium. Sequencing coverage bias, incomplete penetrance, and variable phenotype severity may account for this discrepancy in genotype–phenotype correlation.

Teleost genome duplication resulted in many mammalian genes being represented by two variants in fish, which often serve non-identical functions. Indeed, while *fhl1a* was reported to be expressed and regulates muscular and cardiac function in fish, we noted that large scale in-situ hybridization experiments pointed towards strong and specific expression *fhl1b* in the CS, which functions as the main calcium-regulating hormone in fish. CS produce and secrete PTH1 and STC-1 which act as the main hyper- and hypocalcemic hormones, respectively. Bioinformatic gene interaction analysis revealed that Fhl1 interacts with many calcium-regulatory proteins. Next, through the use of Fhl1b MO, we were able to demonstrate a role for *fhl1b* in calcium regulation in fish, which exhibited aberrantly high calcium levels in response to *fhl1b* downregulation. Gene expression assays revealed that this effect is probably linked to enhanced PTH1 expression in *fhl1b* morphants. While these results establish a direct role for *fhl1b* in calcium regulation in fish, this phenotype is clearly opposite than that seen in the patient. Nonetheless, it should be noted that calcium metabolism in mammals and fish is significantly different, reflecting not only the evolutionary distance but also the different environments in which the two organisms function. In particular, fish rely on calcium influx via the skin and gills as the main source of calcium, whereas humans depend on dietary calcium and bone metabolism to increase circulating calcium levels. Indeed, when we over-expressed *FHL1b* in human cells, we observed strong and significant increase in activation of the human *PTH* gene, confirming that FHL1 loss of function in the patient might well be the etiologic factor leading to hypoparathyroidism.

Of note, *fhl1b* was recently demonstrated to play a major role in organ differentiation in fish, where it has been shown to dictate the pancreas–liver fate decision during development. Therefore, it is possible that FHL1 could regulate not only PTH expression but also the development of the parathyroid gland. In fact, while this obviously would require further experimentation to prove, we propose that *FHL1* might be the disease-causing gene of XLHPT.

In summary, we have uncovered FHL1 as a novel potential regulator of calcium homeostasis in both fish and humans and have implicated it in isolated hypoparathyroidism. This effect is probably mediated by the control over PTH expression and potentially over parathyroid development. While the exact mechanism linking FHL1 to PTH still needs to be elucidated, this report contributes another important layer to the calcium-regulatory network.

## Materials and methods

### Library preparation, exome capture, and sequencing

Genomic DNA was isolated from peripheral blood leukocytes. Library preparation for NGS was performed according to the TruSeq (Illumina) sample-preparation protocol. DNA libraries were then hybridized to exome-capture probes with NimbleGenSeqCap EZ Human Exome Library, version 2.0 (Roche NimbleGen). Exome-enriched libraries were sequenced on the HiSeq 2000 (Illumina) with an average coverage of 80-fold per each sample.

### Variant calling and prioritization

Sequence reads were aligned to the reference human genome (GRCh37/hg19), using the Burrows Wheeler Aligner (BWA) (Li and Durbin 2009). Variants were called following the Genome Analysis Toolkit (GATK) (McKenna et al. 2010) best practices. Briefly, duplicate reads were marked using Picard (http://picard.sourceforge.net). Reads were realigned around detected insertions and deletions (indels) and base qualities recalibrated using GATK. Variant calling was performed using the GATK UnifiedGenotyper tool. Variants with low base or mapping qualities, demonstrating strand bias, aberrant read position distribution, or reference vs. alternate quality score discrepancy, were marked and filtered from subsequent analysis. Variants were annotated using ANNOVAR (Wang et al. 2010) with frequency information gathered from dbSNP138 (Sherry et al. 2001), European and general population from the 1000 genomes project (http://www.1000genomes.org/), NHLBI Exome Sequencing Project (http://evs.gs.washington.edu/EVS/), and a personal database of one hundred Ashkenazi Jews exomes. Insertions and deletions (Indels) found adjacent to homopolymers longer than five bases or repeats were marked and were not considered as true candidates. For monogenic diseases diagnosis, variants with allele frequencies higher than 1% in any of the databases were considered prevalent and were excluded from downstream analysis. For polygenic diseases and pharmacologic-related variants, variants with frequency higher than 10% were excluded. Variant severity was predicted using ten different prediction tools (Supplementary Table 3) gathered by dbNSFP (Liu et al. 2013). A variant was considered to be deleterious if more than half of the prediction tools mark it as such. Variants were prioritized by combining the aforementioned annotations with information regarding their affected gene. Gene disease associations were collected from OMIM (http://omim.org/), Orphanet (http://www.orpha.net) and the Human Phenotype Ontology (Robinson and Mundlos 2010). Tissue expression and developmental stages were collected from Uniprot knowledge base (Magrane and Consortium 2011). Genic intolerance to functional variation (RVIS) were retrieved from (Petrovski et al. 2013). Combination of variant and gene annotation data was performed using in-house scripts. Coverage was calculated using the Ensembl coding sequence regions. CNV analysis from exome data was not performed, since current tools have very low recall, low reproducibility, and high error rate (Hong et al. 2016; Kadalayil et al. 2015).

### Sanger validation

*FHL1* variant was tested for all family members via Sanger sequencing with 100% concordance noted between Sanger results and exome analysis.

### Induced network and gene ontology analysis

ConsensusPathDB (Kamburov et al. 2011), which agglomerated the contents of 32 major public repositories for human molecular interactions of heterogeneous types including Gene Ontology, pathway annotations, and protein–protein interaction (PPI) network was used for functional analysis and correlation of gene function. Relevant data integrated for all input genes or a prioritized subset of molecules and mapped onto interaction networks.

### Experimental animals

Zebrafish were reared in local tap water at 28.5 °C under a 14:10-h light–dark photoperiod at the Sheba Cancer Research Center, Ramat Gan, Israel. Experimental protocols were approved by the Tel Aviv University Institutional Animal Care and Utilization Committee.

### Whole-body Ca^2+^ content

The clean embryo medium of 6dpf embryos pool (~20 embryos) was used for calcium extraction using nitric acid as previously described (Tseng et al. 2009). CaCl_2_ was used to generate the standard curves. We calibrated the concentration of environmental calcium that will not lead to increase in blood calcium in normal fish (25 μg/ml) and tested for linear correlation between the number of fish in pool to the total Ca^2+^ measured from their grouped extraction (Supplementary Figs. 1 and 2).

### RNA extraction and RT-PCR

6dpf embryos pool (~20 embryos) were digested with 50 μl trypsin (0.17%) for 60 min in 37C followed by phase separation using chloroform and precipitation using isopropanol. Finally, RNA was washed with 80% of cold ethanol and resuspended in DEPC-treated water. The final RNA concentration and purity were measured using a NanoDrop Spectrophotometer (ND-1000; Thermo Scientific, USA).

Reverse transcription reaction for mRNA was done using random-primer and SuperScript III reverse transcriptase (Invitrogen). SYBR green PCR master mix (Applied Biosystems) was used for qPCR reaction using StepOnePlus real-time PCR system (Applied Biosystems). Expression values are calculated based on the comparative threshold cycle (Ct) method. mRNA expression levels are normalized to B-actin

### Luciferase reporter assay

HEK 293 cells (1 × 105 cells/ml) were seeded into 96-well Opti-plates (Packard Instrument, Inc., Meriden, CT). After overnight incubation, the cells were transfected with PTH::luciferase construct and empty vector/CASR/fhl1b/CASR + Fhl1/FHL1 siRNA/control siRNA. Transfection was performed using Lipofectamine 2000 transfection reagent (Invitrogen) according to the manufacturer’s instructions. After 24 h, the cells were incubated with normal/high Ca^2+^ for 1 or 3 h. Luciferase activities were determined using the Dual Luciferase kit (Promega) according to the manufacturer’s recommendations. Results were normalized to the constitutively expressed Renilla luciferase.

### Microinjection of antisense morpholino oligonucleotides (MOs)

The morpholino oligonucleotide (MO) was obtained from Gene Tools (Philomath, OR). The *fhl1b* morpholino (5′-TTGGACCGGCTTGCCATAGTCAGTC-3′) was prepared with 1 × Danieau solution [in mM: 58 NaCl, 0.7 KCl, 0.4 MgSO_4_, 0.6 Ca(NO_3_)2, 5.0 HEPES pH 7.6]. Uninjected fish were used as the control. *fhl1b* MO (0.3 M) was injected to zebrafish embryos at one cell stage. No significant morphological phenotype was found.

## Electronic supplementary material

Below is the link to the electronic supplementary material.

Supplementary material 1 (DOCX 252 kb)
