# Supplementary material for: A rare variant in the FHL1 gene associated with X-linked recessive hypoparathyroidism
Source: Hum Genet. 2017 Apr 25;136(7):835–45. doi: 10.1007/s00439-017-1804-9 (PMC5487855; doi:10.1007/s00439-017-1804-9)
Supplement: Supplementary file 1 — Supplementary material 1 (DOCX 252 kb) [file 439_2017_1804_MOESM1_ESM.docx]

**Supplementary Material, Pillar et al., A novel variant in the FHL1 gene associated with X-linked recessive hypoparathyroidism**

Supplementary Table 1: Gene Ontology enrichment of FHL1 and hypocalcemia related genes

| ID | Description | GeneRatio | geneID |
| --- | --- | --- | --- |
| GO:0030154 | cell differentiation | 5\|16 | TBX1/GNA11/GATA3/GNAS/FHL1 |
| GO:0034220 | ion transmembrane transport | 8\|16 | SLC34A1/SLC34A2/SLC20A1/SLC20A2/SLC17A1/SLC17A4/TRPM6/FHL1 |
| GO:0048731 | system development | 8\|16 | PTH/SLC34A1/TBX1/GCM2/GNA11/GATA3/GNAS/FHL1 |
| GO:0009887 | organ morphogenesis | 5\|16 | SLC34A1/TBX1/GATA3/GNAS/FHL1 |
| GO:0048513 | organ development | 8\|16 | PTH/SLC34A1/TBX1/GCM2/GNA11/GATA3/GNAS/FHL1 |
| GO:0006811 | ion transport | 9\|16 | SLC34A1/SLC34A2/SLC20A1/SLC20A2/SLC17A1/SLC17A4/CASR/TRPM6/FHL1 |
| GO:0055085 | transmembrane transport | 9\|16 | SLC34A1/SLC34A2/SLC20A1/SLC20A2/SLC17A1/SLC17A4/GNAS/TRPM6/FHL1 |
| GO:0051050 | positive regulation of transport | 4\|16 | PTH/SLC34A1/GATA3/FHL1 |
| GO:0044765 | single-organism transport | 12\|16 | PTH/SLC34A1/SLC34A2/SLC20A1/SLC20A2/SLC17A1/SLC17A4/CASR/GATA3/GNAS/TRPM6/FHL1 |
| GO:0051049 | regulation of transport | 5\|16 | PTH/SLC34A1/GATA3/GNAS/FHL1 |
| GO:0048523 | negative regulation of cellular process | 4\|16 | PTH/TBX1/GATA3/FHL1 |
| GO:0048878 | chemical homeostasis | 7\|16 | PTH/SLC34A1/SLC34A2/GCM2/CASR/GNAS/FHL1 |

Supplementary Table 2: Genes expressed in the corpuscle of stannius

| NCBI Reference Sequence | Gene symbol | Gene name | Expression |
| --- | --- | --- | --- |
| NM_001045457 | *STC1* | Stanniocalcin1 | Restricted to corpuscles of Stannius |
| NM_131211 | *GATA3* | GATA-binding protein 3 | - Protruding-mouth chondrocranium cartilage - cranial neural crest, - corpuscles of Stannius - otic vesicle - macula - pharyngeal pouch - pronephric duct |
| NM_207078 | *FAM49bb* | Family with sequence similarity 49, Member Bb | - corpuscles of Stannius - forebrain - pronephric duct |
| NM_199217 | *FHL1b* | Four and a Half LIM domains 1b | - notochord - pancreas primordium - primitive heart tube - pronephric duct - corpuscles of Stannius |

Supplementary Table 3: Prediction tools used to set variant pathogenicity

| Severity prediction | SIFT, MutationAssessor, Mutation Taster, GWAVA, PolyPhen2, FATHMM, Silva, LRT |
| --- | --- |
| Conservation prediction | SiPhy, GERP++, PhyloP and PhastCons |

Supplementary Table 4: Primer sequences for RT-PCR

| Gene name | Primer sequence |
| --- | --- |
| CASR | F-5’-TGCTGGGTCTTTTCTACATCC-3’ R-5’CGTCTGTAGGGATTGTCCTCA-3’ |
| ECAC | F-5'-TCCTTTCCCATCACCCTCT-3' R-5'-GCACTGTGGCAACTTTCGT-3' |
| PMCA2 | F-5'-AAGCAGTTCAGGGGTTTAC 3' R-5'-CAGATCATTGCCTTGTATCA-3' |
| NCX1B | F-5'-TAAAGTGGCAGCGATACAGGT-3' R-5'-CAGATCAAGGCGAAGATGG-3’ |
| PTH1 | F- 5’-GTTTCCATCAACGGGAATTT-3’ R-5’-CATCAGCTGCACTTCATTCA-3’ |
| PTH2 | F-5’-ATACGTTGTTTGGAGAAAGCC-3’ R-5’-CATTGTGCATCAGCTGAACTT-3’ |
| STC1 | F-5’-CACGAGATCTGCAACGTCTT-3’ R-5’-AGCGCTTAATGGTCTGGAAC-3’ |
| GATA3 | F-5'- GGATGGCACCGGTCACTATT3' R-5'-CAGCAGACAGCCTCCGTTT-3' |
| FHL1B | F-5'-GACACAAGAGGAGCTTTTCGC-3' R-5'-GCATCGCACACAGACTTGTTT-3' |


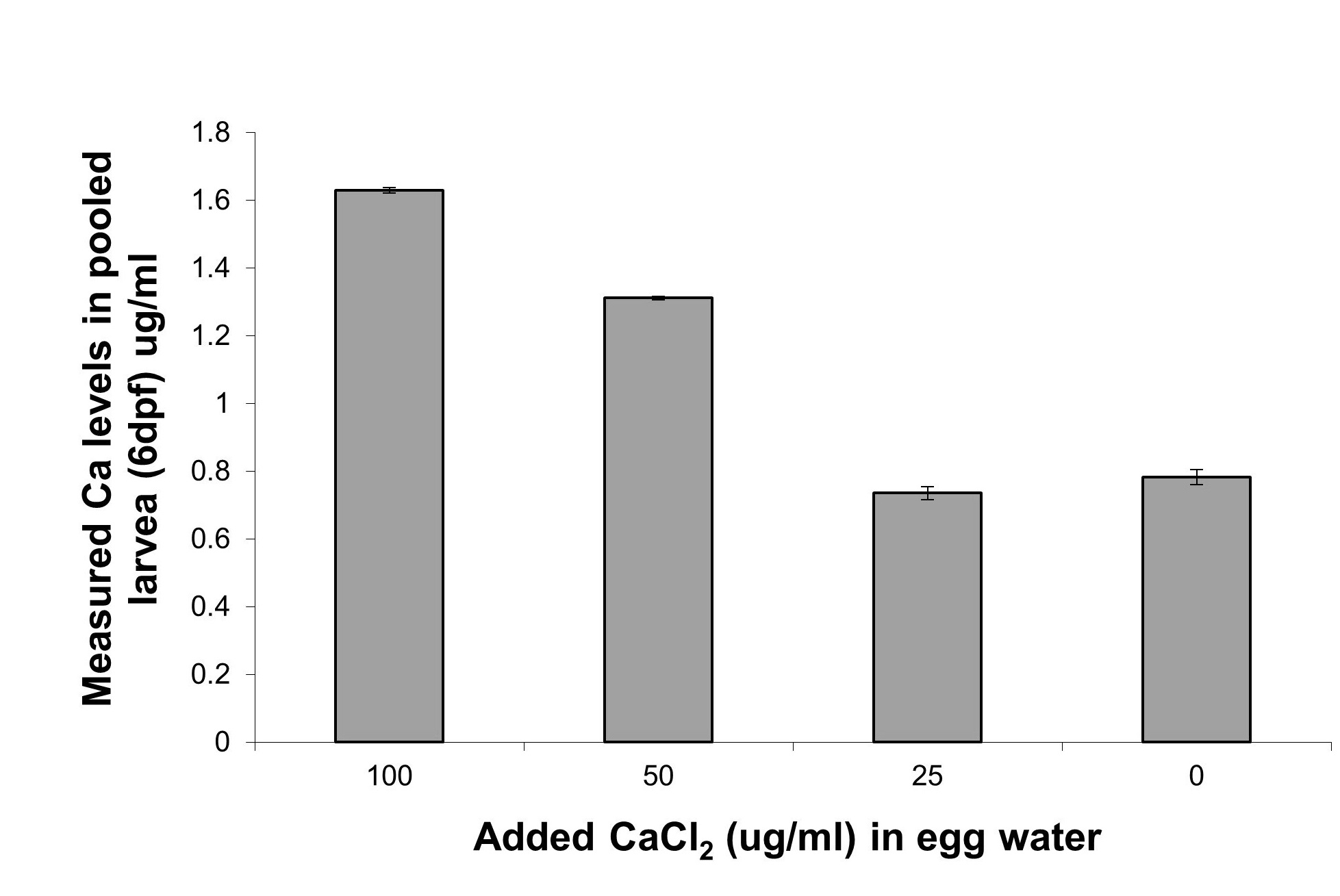


Supplementary Figure 1: Effect of added Ca^2+^ to the measured Ca^2+^ levels in fish. Four CaCl2 concentrations (100ug/ml, 50ug/ml, 25ug/ml and 0) were add into the egg water. Ca^2+^ was measured on larvae 6dpf.


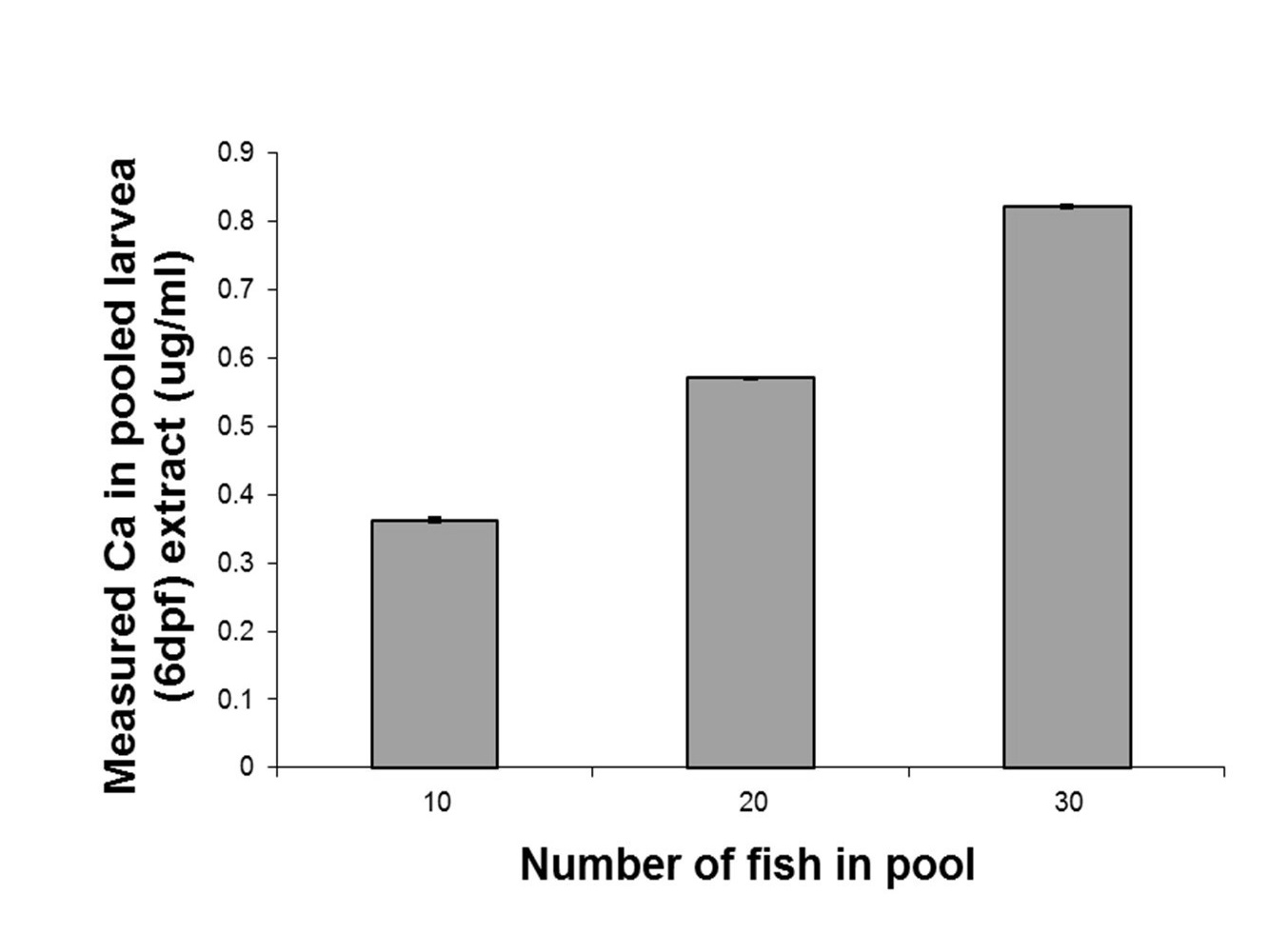


Supplementary Figure 2: Change in Ca^2+^ measured as an effect of number of fish in pool.
